# Renal Chemerin Expression is Induced in Models of Hypertensive Nephropathy and Glomerulonephritis and Correlates with Markers of Inflammation and Fibrosis

**DOI:** 10.3390/ijms20246240

**Published:** 2019-12-11

**Authors:** Alexander Mocker, Karl F. Hilgers, Nada Cordasic, Rainer Wachtveitl, Carlos Menendez-Castro, Joachim Woelfle, Andrea Hartner, Fabian B. Fahlbusch

**Affiliations:** 1 Department of Pediatrics and Adolescent Medicine, University Hospital of Erlangen, 91054 Erlangen, Germany; alexmocker@t-online.de (A.M.); Carlos.Menendez-Castro@uk-erlangen.de (C.M.-C.); joachim.woelfle@uk-erlangen.de (J.W.); andrea.hartner@uk-erlangen.de (A.H.); 2 Department of Nephrology and Hypertension, University Hospital of Erlangen, 91054 Erlangen, Germany; karl.hilgers@uk-erlangen.de (K.F.H.); Nada.Cordasic@uk-erlangen.de (N.C.); Rainer.Wachtveitl@uk-erlangen.de (R.W.)

**Keywords:** chemerin, CmklR1, 2-kidney-1-clip, 2k1c, Thy1.1 nephritis, renovascular hypertension, renal inflammation, renal injury, renal fibrosis

## Abstract

Chemerin and its receptor, chemokine-like receptor 1 (CmklR1), are associated with chemotaxis, inflammation, and endothelial function, especially in metabolic syndrome, coronary heart disease, and hypertension. In humans, circulating chemerin levels and renal function show an inverse relation. So far, little is known about the potential role of chemerin in hypertensive nephropathy and renal inflammation. Therefore, we determined systemic and renal chemerin levels in 2-kidney-1-clip (2k1c) hypertensive and Thy1.1 nephritic rats, respectively, to explore the correlation between chemerin and markers of renal inflammation and fibrosis. Immunohistochemistry revealed a model-specific induction of chemerin expression at the corresponding site of renal damage (tubular vs. glomerular). In both models, renal expression of chemerin (RT-PCR, Western blot) was increased and correlated positively with markers of inflammation and fibrosis. In contrast, circulating chemerin levels remained unchanged. Taken together, these findings demonstrate that renal chemerin expression is associated with processes of inflammation and fibrosis-related to renal damage. However, its use as circulating biomarker of renal inflammation seems to be limited in our rat models.

## 1. Introduction

The significance of end-stage renal disease (ESRD) remains clinically relevant due to its high mortality and morbidity, as well as the lack of effective preventive therapeutic interventions [1]. ESRD results from different forms of renal injury, e.g., arterial hypertension or glomerulonephritis [2,3]. Alterations in the renal microenvironment trigger pathologic immune cell responses with a subsequent acceleration of progressive renal failure, in the setting of loss of glomeruli, tubular atrophy and fibrosis, with reduced glomerular filtration rate (GFR) [2,4]. Unfortunately, the molecular pathways driving persistent renal inflammation are only partly understood to date. Therefore, further research is warranted regarding the regulatory components of inflammation-induced kidney damage in order to develop targeted therapeutics to prevent ESRD effectively. Chemokines and cytokines are both important regulatory mediators of kidney inflammation and potential therapeutic targets [5,6]. They are produced by resident kidney cells, particularly by podocytes, tubular and mesangial cells, as well as by microvascular endothelial cells [6]. Certain mediator combinations determine the recruitment of specific leukocyte subtypes to sites of renal inflammation [2].

Recently, the adipokine chemerin, also known as tazarotene-induced gene 2 protein (TIG2) or retinoic acid receptor responder protein 2 (RARRES2), was introduced as a novel chemoattractant protein [7]. It acts as a ligand for the G protein-coupled receptor CmklR1, also known as ChemR23, and was found to stimulate chemotaxis of dendritic cells and macrophages to sites of inflammation [7,8]. Beyond its classical role in adipogenesis and adipocyte metabolism [9,10], the potential involvement of chemerin in cardiovascular and renal dysfunction has recently been acknowledged [11]. Chemerin appears to form an integral link in metabolic syndrome, connecting obesity, the related dysfunctional cardiometabolic state, and the associated chronic inflammation of adipose tissue [12].

Several investigations have addressed circulating chemerin levels and their pathophysiologic relevance in cohorts with chronic kidney disease [11]. Unrelated to the method of determination [11], serum creatinine is significantly and independently associated with serum chemerin [13,14]. The level of circulating chemerin has been shown to be dependent on GFR and inversely correlated with renal function. A two-fold increase of serum chemerin has been reported in patients on hemodialysis [15]. These results were strengthened by an investigation of ESRD-patients undergoing kidney transplantation [14], whose elevated serum chemerin levels returned to baseline values observed in healthy controls three months after transplant. Furthermore, elevated chemerin levels persisted in ESRD patients on hemodialysis compared to healthy controls or kidney transplanted patients. Nonetheless, hemodialysis reduced high serum chemerin levels to some extent [16]. With adipose tissue as the main source of circulating chemerin [9], it remains to be determined whether elevated serum chemerin levels are due to the increase of fat mass in metabolic phenotypes or to the related renal damage leading to impaired renal elimination. The latter seems more likely [11], as patients with chronic kidney disease showed no difference in subcutaneous adipose tissue chemerin production at the mRNA level [16]. However, the role of visceral adipose tissue cannot be completely ruled out [11]. Also, beyond its role in chemerin elimination, the kidney itself may influence serum chemerin concentrations via its synthesis, as chemerin expression can be found in animal kidneys [9]. In order to investigate the role of renal chemerin and its potential use as a diagnostic marker of kidney disease, our current study characterized its systemic and local expression in established animal models of hypertensive nephropathy and glomerulonephritis using 2-kidney-1-clip (2k1c) hypertensive and Thy1.1 nephritic rats, respectively.

## 2. Results

### 2.1. Chemerin is Induced in Kidneys Exposed to High Blood Pressure

**Figure 1:**
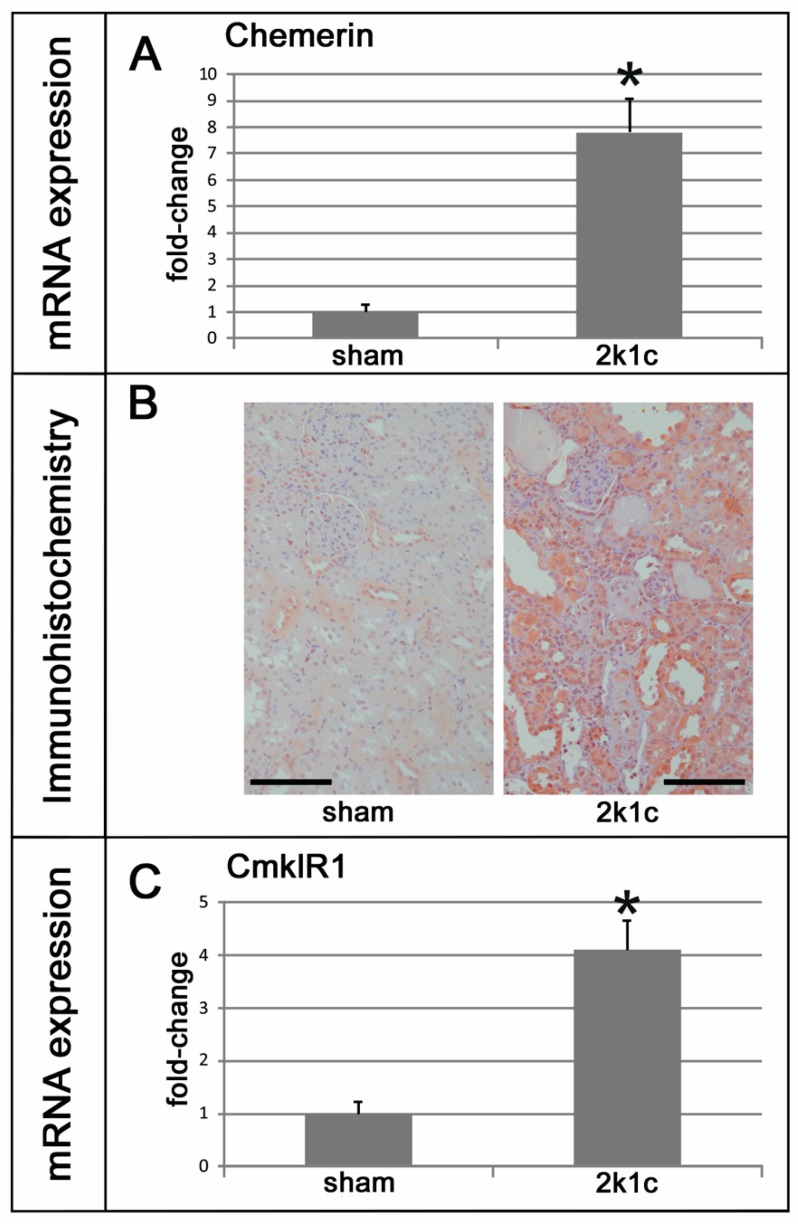
Chemerin and CmklR1 in 2k1c hypertensive nephropathy. (**A**) Chemerin mRNA expression levels in the kidneys of 2k1c hypertensive (2k1c) and control (sham) rats. (**B**) Exemplary photomicrographs of renal tissue from hypertensive (2k1c) and control (sham) rats stained for chemerin. Black bar represents 100 µm. (**C**) CmklR1 mRNA expression levels in the kidneys of 2k1c hypertensive (2k1c) and control (sham) rats. * *p* < 0.05 vs. sham control kidneys.

**Figure 2:**
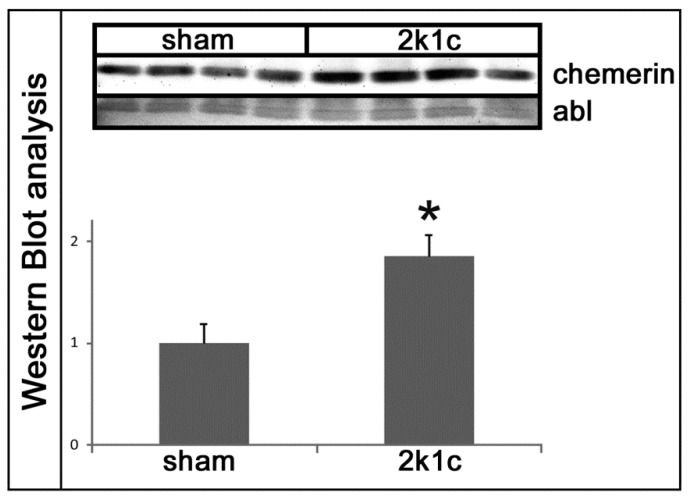
Western blot analysis of chemerin protein expression in the hypertensive kidneys of 2k1c rats. Amido black staining (abl) of the blot served as a loading control. Bar graph: densitometric analysis of Western Blot. * *p* < 0.05 vs. sham control kidneys.

Five weeks after clipping of the left renal artery, the weights of the contralateral right kidneys exposed to high blood pressure were significantly higher than the right kidneys of the sham-operated controls (Table 1). Blood pressure and left ventricular weights of 2k1c rats were increased compared to controls (Table 1). Serum urea and creatinine, as markers of renal damage, were elevated in 2k1c rats compared to controls (Table 1). The expression levels of chemerin were significantly higher in the right kidneys of 2k1c hypertensive animals compared to the kidneys of controls (Figure 1A). Immunohistochemical evaluation of chemerin in kidneys revealed some discrete vascular and distal tubular staining for chemerin in control kidneys, with a prominent increase in chemerin immunoreactivity in the tubulo-interstitium of hypertensive kidneys (Figure 1B). The expression of the chemerin receptor CmklR1 was also induced in hypertensive kidneys (Figure 1C). Moreover, a Western blot analysis revealed an increase in chemerin protein in hypertensive kidneys (Figure 2).

### 2.2. In Kidneys Exposed to High Blood Pressure, Chemerin Expression Correlates with Markers of Renal Damage, Inflammation, and Fibrosis

**Figure 3:**
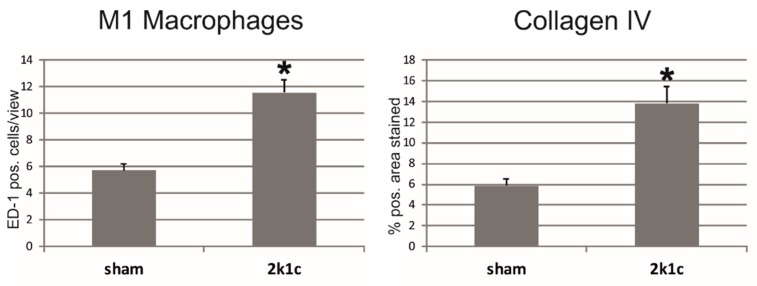
M1 macrophage infiltration and expansion of collagen IV in the tubulointerstitial area of rats with 2k1c hypertensive nephropathy. Sham, control sham operation; ED-1, M1 macrophage marker; * *p* < 0.05 vs. sham control kidneys, data are means ± error of the mean.

Increased infiltration of M1 and M2 macrophages, neutrophil granulocytes, as well as total and helper T-cells, but not of cytotoxic T-cells into the right kidneys of 2k1c rats was observed (Table 2 and Figure 3). Exemplary photomicrographs are shown in Supplementary Figures S1–S3. The expression of TGFβ-1, a central mediator of tissue fibrosis [17], was upregulated in the kidneys of 2k1c rats (Table 3). Moreover, increased smooth muscle actin expression and the presence of more smooth muscle actin positive interstitial cells indicate pronounced fibroblast activation [18] in the right kidney of hypertensive rats (Table 3). Consequently, the expression of the matrix components fibronectin and collagens I, III, and IV was augmented in the right kidneys of these rats (Table 3). The expression of collagens I and IV in right kidney tissue was more prominent in 2k1c rats than in the right kidneys of control rats (Table 3 and Figure 3). Exemplary photomicrographs are shown in Supplementary Figure S4.

**Figure 4:**
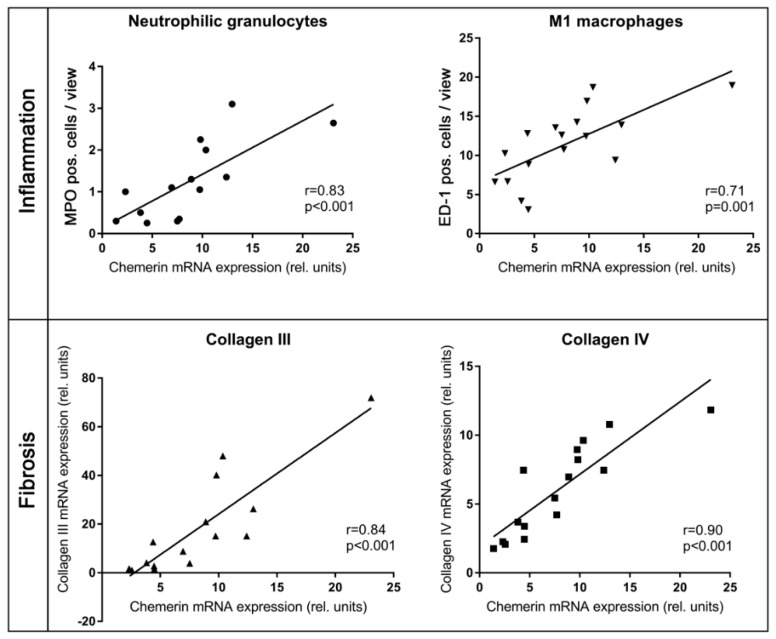
Correlation of chemerin expression with infiltration of neutrophilic granulocytes and M1 macrophages and the expression of collagens III and IV. MPO, myeloperoxidase (marker for neutrophil granulocytes), ED-1, marker for rat M1 macrophages.

The expression of chemerin in the right kidney of hypertensive rats correlated with serum levels of urea and creatinine, but not with blood pressure levels (Table 4). There was a correlation between chemerin expression and M1 macrophage and neutrophil granulocyte infiltration into the right kidneys (Figure 4). Chemerin expression also correlated with fibroblast activation, TGFβ-1 expression and the expression of several matrix molecules (Table 4 and Figure 4). Furthermore, there was a high correlation of chemerin expression with the expression of its receptor CmklR1 (Table 4).

### 2.3. Chemerin is Induced in Glomeruli Afflicted with Thy1.1 Nephritis and Correlates with Markers of Renal Damage, Inflammation, and Fibrosis

**Figure 5:**
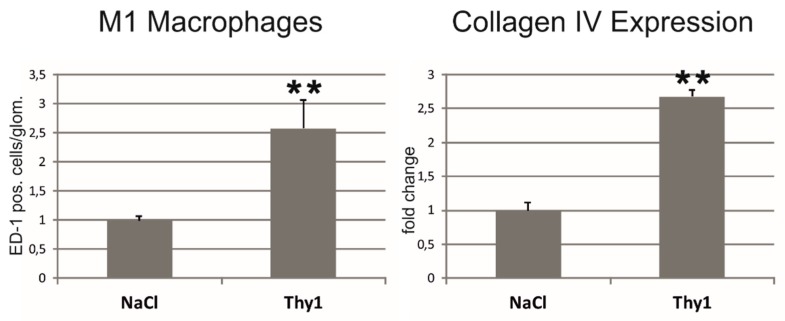
Glomerular M1 macrophage infiltration and collagen IV expression in the renal cortex of rats with anti-Thy1.1 mesangioproliferative glomerulonephritis. NaCl, control vehicle-injected; ED-1, M1 macrophage marker; ** *p* < 0.01; data are means ± error of the mean.

**Figure 6:**
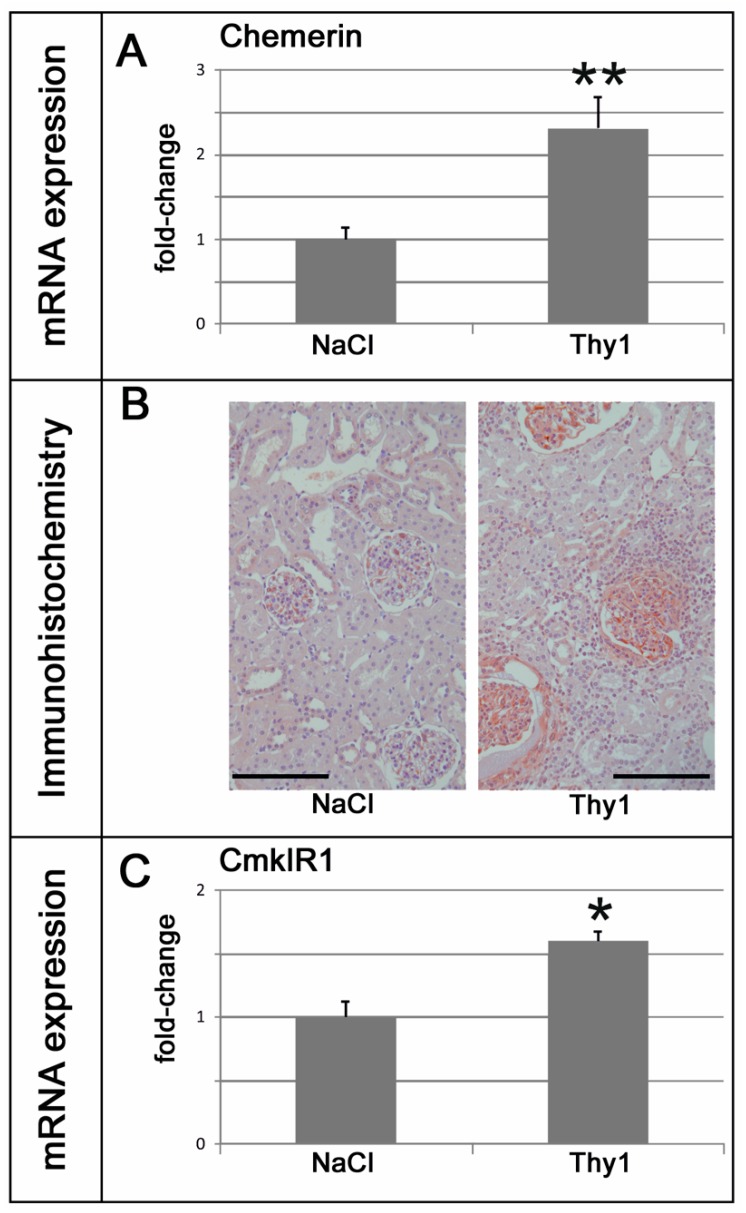
Chemerin and CmklR1 in anti-Thy1.1 glomerulonephritis: (**A**) mRNA expression of chemerin levels in the kidneys of Thy1.1 nephritic (Thy1) and control (NaCl) rats. (**B**) Localization of chemerin protein in renal sections of Thy1.1 nephritic and control rats. Black bar represents 100 µm. (**C**) mRNA expression of the chemerin receptor CmklR1 levels in the kidneys of Thy1.1 nephritic (Thy1) and control (NaCl) rats. NaCl, NaCl infused controls. Thy1, anti-Thy1.1 infused glomerulonephritic animals. ** *p* < 0.01, * *p* < 0.05 vs. control kidneys.

Two weeks after the induction of a Thy1.1 glomerulonephritis, an increase in serum creatinine and albuminuria were observed (Table 5). Glomerular M1 macrophage infiltration and renal collagen IV expression were increased in nephritic kidneys (Figure 5 and Figure S5). Blood pressure was not altered in Thy1.1 nephritic rats (Table 5). Chemerin expression was increased in the renal tissue of Thy1.1 nephritic rats (Figure 6A). Staining for chemerin revealed prominent glomerular immunoreactivity in nephritic glomeruli, while in control glomeruli, only some podocytes stained positive (Figure 6B). The expression of the chemerin receptor CmklR1 was also somewhat increased (Figure 6C).

The expression of chemerin in kidneys with Thy1.1 glomerulonephritis correlated with serum creatinine levels, albuminuria, glomerular infiltration of M1 macrophages, and renal collagen IV expression (Table 6). A correlation between chemerin expression and CmklR1 expression was also detected (Table 6).

### 2.4. Chemerin Plasma Levels are Not Increased in Rat Models of Renal Injury

To clarify whether increases of renal tissue chemerin are also reflected by increases in plasma chemerin, ELISA assays were performed. In 2k1c hypertensive rats, plasma chemerin was not increased compared to sham-operated controls (1.15 ± 0.06 ng/mL in hypertensive rats versus 1.29 ± 0.11 ng/mL in controls). Likewise, in Thy1.1 glomerulonephritic rats, plasma chemerin levels were similar to the plasma levels of control rats (1.13 ± 0.19 ng/mL in nephritic rats versus 1.22 ± 0.10 ng/mL in controls).

## 3. Discussion

We have demonstrated specific induction of local expression patterns of chemerin related to the underlying model of renal injury, i.e., tubular-interstitial (2k1c, in kidneys exposed to high blood pressure) and glomerular damage (Thy1.1 nephritis). In our study, renal chemerin expression positively correlated with markers of renal damage and inflammation in 2k1c hypertensive animals and Thy1.1 nephritic rats, indicating a possible involvement of chemerin in these processes, as seen in adipose tissue [19]. Concomitantly, the expression of the chemerin receptor CmklR1 was also found to be induced in hypertensive kidneys, while somewhat increased in anti-Thy1.1 treated animals. Of the three known chemerin receptors (i.e., chemokine-like receptor 1 (CmklR1), G-protein-coupled receptor (GPR) 1, and C-C motif receptor-like (CCRL) 2, only CmklR1 sufficiently mediates intracellular signaling functions (reviewed by [12]). Besides its expression in hematopoietic tissues, CmklR1 is strongly expressed in cells of the immune system (e.g., blood monocytes, monocyte-derived human macrophages, immature dendritic cells, CD4+ T lymphocytes) [7,20]. CmklR1 directs the migration of immune cells to lymphoid organs and inflamed tissues [21]. Thus, the observed renal damage in our models might have partly resulted from chemerin-induced, CmklR1-mediated chemoattraction of immune cells to the respective sites of renal damage (i.e., tubulo-interstitium in 2k1c model and glomerulus in Thy1.1 nephritic rats).

Interestingly, CmklR1 expression was additionally observed in human microvascular endothelial cells (ECs) by Kaur et al. [22]. Thus, para-/autocrine effects of chemerin on renal vasculature in our model are also possible. Kaur et al. showed that the expression of the receptor was significantly up-regulated by pro-inflammatory cytokines in human ECs and had strong angiogenic potential via activation of PI3K/Akt and MAPK pathways in these cells [22]. The involvement of an imbalance of angiogenesis-related factors in the progression of CKD and the therapeutic potential of modulating these factors in CKD has been acknowledged (reviewed by [23]). Furthermore, Kaur et al. showed that chemerin was able to induce the activity of members of the matrix metalloproteinase (MMP) family in ECs [22], which play an important role in the degradation of the extracellular matrix (ECM) [24]. This effect is also crucial in the development and progression of CKD; however, non-proteolytic functions of MMPs might also play a role [24]. In line with these observations, we found chemerin to correlate with markers of renal fibrosis.

So far, the exact mechanisms of renal chemerin induction in our animals remain unknown. However, a potential role of angiotensin 2 (Ang II) in the regulation of chemerin expression has been proposed by others. Using a model of diabetic nephropathy, Yu et al. [25] were able to show that the expression of chemerin in the kidney of diabetic rats was significantly elevated compared to control animals, suggesting that chemerin might be relevant to the model-specific renal pathology. Treatment with irbesartan (Ang II type 1 receptor antagonist) appeared to reduce the renal chemerin expression in these diabetic animals secondary to a reduction in renin–angiotensin system (RAS) components [25].

The Goldblatt 2k1c rat hypertension model is a long-established and widely employed model in the study of renal artery stenosis and renovascular hypertension [26,27]. We have observed that the RAS, including Ang II, is closely related with the 2k1c model [28] since their levels are elevated in the development and maintenance of hypertension in these animals: Early on, hypertension in 2k1c animals is characterized by increased plasma renin levels in response to low renal arterial pressure and subsequently by an increase in circulating Ang II. Later, hypertension is maintained by a continuously activated RAS, as contralateral pressure diuresis of the unaffected kidney prevents hypervolemia [29,30]. Persistent elevation of Ang II also triggers an inflammatory response, characterized by the infiltration of macrophages (ED-1), tubular overexpression of macrophage chemotactic and adhesion molecules, such as osteopontin (OPN), MCP-1 and the expression of inflammatory cytokines, ultimately aggravating renal damage induced by hypertension [3,31,32]. 

A potential interaction of Ang II with chemerin in the 2k1c model remains to be determined. Ang II is a key mediator of CKD. A blockade of the Ang II type 1 receptor prevents lethal malignant hypertension [28]. It is also understood that Ang II mediates renal fibrosis by stimulating the endogenous synthesis of transforming growth factor-β (TGF-β) [33] in damaged kidney cells, thereby stimulating the synthesis of the extracellular matrix (ECM), and inhibiting the action of MMPs [34]. TGF-β induces the transformation of fibroblasts into myofibroblasts (α-smooth muscle actin-positive cells, α-SMA) and stimulates the expression of fibronectin (FN) and collagen type III (Col III). This induces the development of renal fibrosis, leads to functional deterioration and increases kidney damage [34,35,36]. Notably, we have found that renal chemerin levels correlated positively with these Ang II-dependent markers of inflammation and fibrosis in both our models of renal injury. Moreover, other RAS-associated factors, such as aldosterone/mineralocorticoid receptor [37] or the activity of the angiotensin-converting enzyme (ACE) [38], might be relevant in the function of renal chemerin. 

Similar to 2k1c animals, RAS activation also plays a pivotal role in the progression of glomerulonephritis (GN) in Thy1.1 nephritic rats [39]. Thus, potential cross-talk of chemerin and Ang II [25] at the glomerular level might be conceivable. The immunohistochemically observed glomerular expression of chemerin in our Thy1.1 nephritic rats closely resembled glomerular cyto/chemokine expression patterns typically found in nephritic renal damage [40], thereby underscoring the potential role of chemerin as a damage-site specific chemoattractant. The most commonly used model of selective mesangial cell damage [41,42,43], anti-Thy1.1-induced glomerulonephritis, resembles some human forms of GN [44], where the renal damage is characterized by the continued accumulation of ECM, related to the overproduction of glomerular TGF-β.

As a limitation, the role of Ang II in our 2k1c animals and Thy1.1. nephritic rats remains speculative due to the lack of functional data regarding RAS signaling. Future studies are needed to uncover the mechanistic insights of chemerin signaling transduction pathways in the kidney, with a special focus on the exploration of potential therapeutic targets for renal fibrosis and inflammation.

Despite the association of increased chemerin with (renal) inflammation and fibrosis, Yamamoto et al. found an association of elevated chemerin levels with a survival advantage in dialysis patients [45]. This seems controversial, as CKD induces premature vascular aging with vascular calcification and increased arterial stiffness [46]. In addition, previous studies had indicated a role of CmklR1 for the vascular smooth muscle cell (VSMC) atherosclerotic phenotype [47], characterized by vascular inflammation and intimal hyperplasia [48,49]. Surprisingly, chemerin seemed to inhibit atherogenesis through CmklR1 [48,50]. Carracedo et al. [51] were able to show that chemerin treatment of isolated wild-type mouse VSMCs significantly reduced phosphate-induced calcification and increased expression of the calcification inhibitor matrix-gla-protein (MGP). In contrast, VSMCs of CmklR1 knock-out mice were devoid of these effects. This suggests that elevated chemerin might, in fact, exert a direct protective vascular role in CKD, while negatively altering the local microenvironment via attraction of immune cells at the same time.

In contrast to findings in humans [11], we did not observe an induction of circulating chemerin. So far, the majority of existing human reports focus on elevated circulating levels of chemerin in CKD patients, while little is known about the local renal expression of the protein. Based on our finding of increased chemerin expression in ESRD, one could speculate that this may contribute to the reported increase of circulating chemerin levels in ESRD, apart from the postulated reduced chemerin renal elimination capacity associated with ESRD [14]. However, as we were unable to detect an increase of circulating chemerin levels in both animal models of our study, it remains uncertain to what extent such local changes in our rodent models might translate into a significant increase of circulating chemerin levels observed in human ESRD.

The lack of increased circulating chemerin seemed unrelated to an inoperative experimental design. Clipping of the left renal artery and anti-Thy1.1 treatment both sufficiently reduced kidney function, as determined by a ~2-fold increase in serum urea and creatinine. Also, renal weight was significantly increased in both rodent models. These results match findings previously obtained by our group in 2k1c rats [52]. It is possible that despite the detectable renal affliction of our animals and its correlation with local chemerin expression, the functional renal restriction might not have been relevant enough to fully resemble the level of kidney failure seen in human ESRD [14]. Thus, the renal capacity to eliminate increasing levels of circulating chemerin might still have been sufficient in the examined animal models.

A current methodological limitation in the field of chemerin research is the lack of analysis of the multitude of existing chemerin pre-cursors [12,19]. Chemerin is proteolytically processed (e.g., by cathepsin G, elastase, plasmin, and tryptase) into different active and inactive chemerin peptides, such as pre-prochemerin and mature prochemerin, which might exert specific functions on their own. 

In summary, our findings provide novel evidence that renal chemerin expression in 2k1c and Thy1.1 nephritic rats are associated with markers of kidney inflammation and fibrosis. However, we did not find elevated levels of circulating chemerin in these animals. Thus, chemerin might not serve as a biomarker in these models.

## 4. Materials and Methods

### 4.1. Experimental Procedures

All animal experiments were performed in compliance with the DIRECTIVE 2010/63/EU of the European Parliament and were approved by the local government authorities (Regierung of Mittelfranken, AZ 54-2532.1-51/12, 22 October 2013 and AZ 55.2.2532-2-526, 18 October 2017). All efforts were made to minimize suffering in the animal cohort. Rats were housed in a room maintained at 22 ± 2 °C, exposed to a 12-h dark/light cycle. The animals had unlimited access to standard rodent nutrition and tap water. 

Induction of hypertensive nephropathy: Two-kidney, one-clip renovascular hypertension (2k1c) was induced in male Sprague–Dawley rats (Charles River, Sulzfeld, Germany) weighing 150–170 g by placing a silver clip of 0.2 mm internal diameter around the left renal artery through a flank incision under isoflurane anesthesia as previously described (*n* = 25) [53]. Control animals underwent a sham operation without placement of the clip (*n* = 10). Analgesia was provided post-operatively in all animals, and as needed later on. Five weeks after the clipping of the renal artery, the experiment was terminated, and renal tissue was collected. 

Induction of acute glomerulonephritis: Male Sprague-Dawley rats (150 to 200 g) were obtained from Charles River Deutschland. Anti-Thy1.1 nephritis was induced in uninephrectomized rats by a single intravenous injection of 1 mg/kg body weight anti-Thy1.1 antibody into the tail vein in light isoflurane anesthesia. Controls received solvent only (*n* = 5 per group). The monoclonal antibody against Thy1.1 (ER4) was from Antibody Solutions (Santa Clara, CA, USA). Anti-Thy1.1 nephritis is an acute mesangioproliferative glomerulonephritis with mesangial expansion and glomerulosclerosis peaking at days 7 to 14 of disease [42,54]. On day 13, animals were housed in metabolic cages for 24 h to collect urine. Five animals per group were sacrificed on day 14 after induction of nephritis and renal tissue was obtained for further evaluation. 

### 4.2. Blood Pressure Measurements

At the end of the experiment, rats were weighed and instrumented with femoral artery catheters for intraarterial blood pressure measurements in anesthesia, as described previously [55]. Measurements were performed on the same day after termination of anesthesia and a recovery phase of 2 h in conscious animals via transducers connected to a polygraph (Hellige, Freiburg, Germany).

### 4.3. Measurement of Serum and Urine Parameters

For urine collection, anti-Thy1.1 nephritic animals were put in metabolic cages for 24 h on the day before sacrifice. Albumin excretion was assessed by enzyme-linked immunosorbent assay (Bethyl Laboratories, Biomol, Hamburg, Germany). For serum analysis, blood was collected from catheters. Thereafter, rats were euthanized by bleeding in deep anesthesia. Plasma creatinine and plasma urea were analyzed using an automatic analyzer Integra 1000 (Roche Diagnostics, Mannheim, Germany). Plasma chemerin was determined using a commercially available ELISA kit (MyBiosource, Biozol, Eching, Germany) according to the manufacturer’s protocol.

### 4.4. Tissue Sampling

After organ weighing, kidneys were decapsulated. Both poles of each kidney and the apical tip of the left ventricle were immediately snap-frozen on liquid nitrogen for protein or RNA extraction. One 6 mm slice of the kidney was put in paraformaldehyde solution (for detection of chemerin), while another 6 mm slice of the remaining kidney was put in methyl-Carnoy solution (60% methanol, 30% chloroform and 10% glacial acetic acid) for fixation. After overnight fixation, tissues were dehydrated by bathing in increasing concentrations of alcohol and embedded in paraffin. Three µm sections were cut with a Leitz SM 2000 R microtome (Leica Instruments, Nussloch, Germany).

### 4.5. Immunohistochemistry

Tissue was processed as described [56]. Immunohistochemical detection of chemerin, collagen I, collagen IV, α-smooth muscle actin (SMA), ED-1, myeloperoxidase (MPO), CD3, CD4, CD8a, and CD163 was performed in methyl Carnoy-fixed tissue sections. Antibodies used are described in Supplementary Table S1. The specificity of the chemerin antibody was confirmed by staining in control tissue: rat skin, lung and testes (see Supplementary Figure S6). Interstitial collagens I and IV were quantified in 30 medium-power views (magnification x200) by means of an 11 × 11-point grid or by densitometric analysis using MetaVue software (Molecular Devices, Sunnyvale, CA, USA). The percentage of grid points corresponding with a stained area or the percentage of stained area in relation to the total area was calculated. SMA, ED-1, MPO, CD3, CD4, CD8a, and CD163 positive cells were counted in 20 medium-power cortical views. All histological evaluations were done by a single investigator blinded to the group assignment.

### 4.6. Western Blot Analysis

Frozen renal tissue was homogenized, protein samples were prepared as described [57] and separated on a denaturing SDS-PAGE gel [58]. After electrophoresis, the gels were electroblotted onto PVDF membranes (Hybond-P, GE Amersham, Munich, Germany), blocked with Rotiblock (Roth, Karlsruhe, Germany) for 1 h and incubated overnight with a primary antibody to chemerin. Protein bands were visualized with secondary horseradish peroxidase-conjugated IgG antibodies (Santa Cruz Biotechnology, 1:50,000), using the Pierce ECL+ system (Thermo Fisher Scientific, Waltham, MA, USA). Blots were quantified using a luminescent imager (LAS-1000, Fujifilm, Berlin, Germany) and Aida 2.1 image analysis software (Raytest, Berlin, Germany). Loading of the blot was quantified by Amido Black staining solution (Sigma, Taufkirchen, Germany).

### 4.7. Real-Time Polymerase Chain Reaction (PCR) Analyses

Renal tissue was homogenized in RLT buffer reagent (Qiagen, Hilden, Germany) with an ultraturrax for 30 s, total RNA was extracted from homogenates by RNeasy Mini columns (Qiagen) according to the manufacturer’s protocol, and real-time RT-PCR was performed [59]. First-strand cDNA was synthesized with TaqMan reverse transcription reagents (Applied Biosystems, Darmstadt, Germany) using random hexamers as primers. Reactions without Multiscribe reverse transcriptase were used as negative controls for genomic DNA contamination. PCR was performed with a StepOnePlus™ sequence detector system (Applied Biosystems, Darmstadt, Germany) and TaqMan or SYBR Green Universal PCR master mix (Applied Biosystems), as described previously [57]. All samples were run in duplicates. Specific mRNA levels in hypertensive animals relative to sham-operated controls were calculated and normalized to a housekeeping gene (18S) with the ∆∆Ct method as specified by the manufacturer (Applied Biosystems). Primer pairs used for experiments are shown in Supplementary Table S2.

### 4.8. Statistical Analysis

Data are expressed as mean ± standard error of the mean (SEM). After testing for normality distribution using Shapiro–Wilk’s test, we performed Student’s *t*-test or the Mann–Whitney U-test, where appropriate. A *p*-value < 0.05 was considered significant. To assess correlations between chemerin and markers of inflammation and fibrosis, Spearman’s correlation coefficients (Spearman’s rho) were calculated. Calculations were carried out using the SPSS 19 software (IBM, Ehningen, Germany) and GraphPad Prism 7.00 (GraphPad Software, La Jolla, CA, USA).

## Figures and Tables

**Table 1:** Physiological parameters of 2k1c experimental groups.

Physiological Parameter	Sham	2k1c	*p*-Value
**Rel. right kidney weight** (mg/g body weight)	3.21 ± 0.06	4.87 ± 0.25	<0.001
**Serum urea** (mg/dL)	37.74 ± 1.13	80.92 ± 11.26	<0.001
**Serum creatinine** (mg/dL)	0.195 ± 0.007	0.335 ± 0.038	<0.001
**Rel. left ventricular weight** (mg/g body weight)	2.05 ± 0.04	3.03 ± 0.21	0.003
**Mean arterial blood pressure** (mm Hg)	113.2 ± 2.6	203.7 ± 5.0	0.014

Sham, sham-operated control group; 2k1c, hypertensive group. Data are means ± standard error of the mean. *p* < 0.05 2k1c versus sham was considered significant.

**Table 2:** Inflammatory cell infiltration in the kidneys of 2k1c.

Cell Type	Sham	2k1c	*p*-Value
**M2 macrophages** (CD163 pos. cells/cortical view)	0.09 ± 0.04	0.86 ± 0.20	<0.001
**Neutrophil granulocytes** (myeloperoxidase pos. cells/cortical view)	0.53 ± 0.15	1.49 ± 0.30	0.039
**Total T-cells** (CD3 pos. cells/cortical view)	1.23 ± 0.47	4.88 ± 1.81	0.046
**Helper T-cells** (CD4 pos. cells/cortical view)	11.56 ± 3.37	75.45 ± 14.60	0.002
**Cytotoxic T-cells** (CD8a pos. cells/cortical view)	3.12 ± 0.38	4.76 ± 0.35	0.456

Sham, sham-operated control group; 2k1c, hypertensive group. Data are means ± standard error of the mean. *p* < 0.05 2k1c versus sham was considered significant.

**Table 3:** Markers of renal fibrosis in 2k1c.

Fibrotic Marker	Sham	2k1c	*p*-Value
**TGFβ-1 expression** (fold change)	1.00 ± 0.25	3.69 ± 0.44	0.001
**Smooth muscle actin expression** (fold change)	1.00 ± 0.21	5.89 ± 0.96	0.014
**Activated fibroblasts** (smooth muscle actin pos. cells/cortical view)	0.26 ± 0.04	5.74 ± 1.50	0.002
**Fibronectin expression** (fold change)	1.00 ± 0.24	8.81 ± 1.55	0.003
**Collagen I expression** (fold change)	1.00 ± 0.32	4.42 ± 0.78	0.024
**Collagen I stain** (% pos. cells/cortical view)	4.66 ± 0.44	7.98 ± 1.14	0.001
**Collagen III expression** (fold change)	1.00 ± 0.49	18.25 ± 5.32	0.004
**Collagen IV expression** (fold change)	1.00 ± 0.22	6.03 ± 0.83	0.001

Sham, sham-operated control group; 2k1c, hypertensive group. Data are means ± standard error of the mean. *p* < 0.05 2k1c versus sham was considered significant.

**Table 4:** Correlation of markers of renal damage, inflammation, and fibrosis with chemerin expression in 2k1c.

Chemerin (mRNA Expression)	r	*p*-Value
**Serum creatinine** (mg/dL)	0.62	**0.009**
**Serum urea** (mg/dL)	0.77	**<0.001**
**Mean arterial blood pressure** (mm Hg)	0.42	0.12
**M1 macrophages** (ED-1 pos. cells/view)	0.71	**0.001**
**M2 macrophages** (CD163 pos. cells/view)	0.51	0.16
**Total T-cells** (CD3 pos. cells/view)	0.27	0.40
**Cytotoxic T-cells** (CD8a pos. cells/view)	0.47	0.11
**Helper T-cells** (CD4 pos. cells/view)	−0.14	0.63
**Neutrophil granulocytes** (MPO pos. cells/view)	0.83	**<0.001**
**Activated myofibroblasts** (SMA-pos. cells/view)	0.74	**0.003**
**Smooth muscle actin** (mRNA expression)	0.65	**0.005**
**Fibronectin** (mRNA expression)	0.86	**<0.001**
**Collagen I** (% pos. area stained)	0.65	**0.043**
**Collagen I** (mRNA expression)	0.83	**<0.001**
**Collagen III** (mRNA expression)	0.84	**<0.001**
**Collagen IV** (% pos. area stained)	0.64	**0.014**
**Collagen IV** (mRNA expression)	0.90	**<0.001**
**TGFβ-1** (mRNA expression)	0.73	**0.001**
**CmklR1** (mRNA expression)	0.89	**<0.001**

r = Spearman–Rho correlation coefficient r, statistical significance was defined as *p*-value < 0.05.

**Table 5:** Markers of renal damage and blood pressure in Thy1 glomerulonephritis.

Damage Marker	NaCl	Thy1	*p*-Value
**Rel. right kidney weight** (mg/g body weight)	4.30 ± 0.15	8.57 ± 0.18	<0.01
**Albuminuria** (mg/24 h)	0.80 ± 0.19	725.79 ± 303.13	<0.01
**Serum creatinine** (mg/dL)	0.19 ± 0.01	0.34 ± 0.03	<0.01
**Cytotoxic T-cells** (CD8a pos. cells/cortical view)	3.51 ± 0.56	5.72 ± 0.41	n.s.
**Helper T-cells** (CD4 pos. cells/cortical view)	3.05 ± 0.61	6.23 ± 2.39	n.s.
**Mean arterial blood pressure** (mm Hg)	118.5 ± 1.5	123.7 ± 6.3	n.s.
**Rel. left ventricular weight** (mg/g body weight)	2.23 ± 0.11	2.49 ± 0.09	n.s.

NaCl, NaCl infused control group; Thy1, glomerulonephritic group. Data are means ± standard error of the mean. *p* < 0.05 2k1c versus sham was considered significant.

**Table 6:** Correlation of markers of renal damage, inflammation and fibrosis with chemerin expression in Thy1 glomerulonephritis.

Chemerin (mRNA Expression)	r	*p*-Value
**CmklR1** (mRNA expression)	0.89	**0.001**
**Serum creatinine** (mg/dL)	0.73	**0.017**
**Albuminuria** (mg/24h)	0.73	**0.016**
**Mean arterial blood pressure** (mm Hg)	0.42	0.262
**M1 macrophages** (ED-1 pos. cells/view)	0.72	**0.019**
**Cytotoxic T-cells** (CD8a pos. cells/view)	0.58	0.082
**Helper T-cells** (CD4 pos. cells/view)	0.39	0.266
**Collagen IV** (mRNA expression)	0.83	**0.005**

r = Spearman–Rho correlation coefficient r, statistical significance was defined as *p*-value < 0.05.

## Supplementary Materials

Supplementary Materials can be found at https://www.mdpi.com/1422-0067/20/24/6240/s1. Figure S1: Exemplary photomicrographs of kidneys with 2k1c nephropathy and control kidneys (sham) stained for the M1 macrophage marker ED1 and the M2 macrophage marker CD163. Figure S2: Exemplary photomicrographs of kidneys with 2k1c nephropathy and control kidneys (sham) stained for the neutrophil marker myeloperoxidase and the T-cell marker CD3. Figure S3: Exemplary photomicrographs of kidneys with 2k1c nephropathy and control kidneys (sham) stained for the T-helper cell marker CD4 and the cytotoxic T-cell marker CD8a. Figure S4: Exemplary photomicrographs of kidneys with 2k1c nephropathy and control kidneys (sham) stained for collagen I and collagen IV. Figure S5: Exemplary photomicrographs of kidneys with Thy1 induced glomerulonephritis and control kidneys (NaCl) stained for the M1 macrophage marker ED1. Figure S6. Specificity testing of the chemerin antibody. The antibody stained skin (Luangsay S et al. 2009, J Immunol 183; Vermi W et al. 2005, J Exp Med 201), lung (Luangsay S et al. 2009, J Immunol 183) and testes (Li L et al. 2014, J Endocrinol 220), as described before. Black arrowheads point to the chemerin positive keratinocyte layer in a skin sample, to the chemerin positive ciliated epithelium of the lung bronchioles and to chemerin positive Leydig cells in testes. Table S1. Antibodies used for immunohistochemistry. Table S2. Primer sequences.
