# Supplementary material for: Renal Chemerin Expression is Induced in Models of Hypertensive Nephropathy and Glomerulonephritis and Correlates with Markers of Inflammation and Fibrosis
Source: Int J Mol Sci. 2019 Dec 11;20(24):6240. doi: 10.3390/ijms20246240 (PMC6941130; doi:10.3390/ijms20246240)
Supplement: Supplementary file 1 [file ijms-20-06240-s001.zip › Supplementary table S1.docx]

**Supplementary table S1: Antibodies used for immunohistochemistry**

| **Antibody** | **Clone** | **Company** | **Dilution** |
| --- | --- | --- | --- |
|  |  |  |  |
| α-smooth muscle actin | 1A4 | DAKO | 1:50 |
| Chemerin |  | Thermo Fisher | 1:100 |
| CD3 | SP7 | abcam | 1:50 |
| CD4 | D7D2Z | Cell Signaling | 1:50 |
| CD8a | OX-8 | abcam | 1:100 |
| CD68 | ED-1 | AbD Serotec | 1:250 |
| CD163 | EPR19518 | abcam | 1:50 |
| Collagen I |  | Biogenesis | 1:50 |
| Collagen IV |  | Southern Biotechnology | 1:100 |
| Myeloperoxidase |  | abcam | 1:50 |
| Osteopontin |  | Santa Cruz Biotechnology | 1:50 |
