# Supplementary material for: Renal Chemerin Expression is Induced in Models of Hypertensive Nephropathy and Glomerulonephritis and Correlates with Markers of Inflammation and Fibrosis
Source: Int J Mol Sci. 2019 Dec 11;20(24):6240. doi: 10.3390/ijms20246240 (PMC6941130; doi:10.3390/ijms20246240)
Supplement: Supplementary file 1 [file ijms-20-06240-s001.zip › Supplementary table S2.docx]

**Supplementary table S2: List of primers pairs and probes used in the study.**

|  | **forward** | **reverse** |
| --- | --- | --- |
| **18S** | 5’- TTG ATT AAG TCC CTG CCC TTT GT -3’ | 5’- CGA TCC GAG GGC CTC ACT A -3’ |
| **α-SMA** | 5’- TCCTGACCCTGAAGTATCCGATA -3’ | 5’- GGTGCCAGATCTTTTCCATGTC -3’ |
| **CCL-2 (MCP-1)** | 5’- CCTCCACCACTATGCAGGTCTC -3’  5’- TCACGCTTCTGGGCCTGTTGTTCA -3’ [Probe] | 5’- GCACGTGGATGCTACAGGC -3’ |
| **Chemerin** | 5’- AAATGGGAGGAAGCGGAAAT -3’ | 5’- CCATCCGGCCTAGAACTTTACC -3’ |
| **CmklR1** | 5’-AAGAGATGGAGTACGAGGGTTACAA -3’ | 5’-GATGTAGTCCGAGCCGTCAGA -3’ |
| **Coll I** | 5’- AGAGCGGAGAGTACTGGATCGA -3’ | 5’- CTGACCTGTCTCCATGTTGCA -3‘ |
| **Coll III** | 5’- ATATCAAACACGCAAGGC -3’ | 5’-GATTAAAGCAAGAGGAACAC -3’ |
| **Coll IV** | 5’- AACGAAAGGGACACGAGGA -3’ | 5’- GGCCAGGAATACCAGGAAGT -3’ |
| **FN** | 5’- TTGCAACCCACCGTGGAGTATGTG -3’ | 5’- CTCGGTAGCCAGTGAGCTTAACAC -3’ |
| **OPN** | 5’- AAAGTGGCTGAGTTTGGCAG -3’  5’- TCAGAGGAGAAGGCGCATTACAGCA -3’ [Probe] | 5’- AAGTGGCTACAGCATCTGAGTGT -3’ |
| **TGFβ-1** | 5’- TGGAAGTGGATCCACGCGCCCAAGG -3’ | 5’- GCAGGAGCGCACGATCATGTTGGAC -3’ |
