# Supplementary figures and images for: Renal Chemerin Expression is Induced in Models of Hypertensive Nephropathy and Glomerulonephritis and Correlates with Markers of Inflammation and Fibrosis
Source: Int J Mol Sci. 2019 Dec 11;20(24):6240. doi: 10.3390/ijms20246240 (PMC6941130; doi:10.3390/ijms20246240)

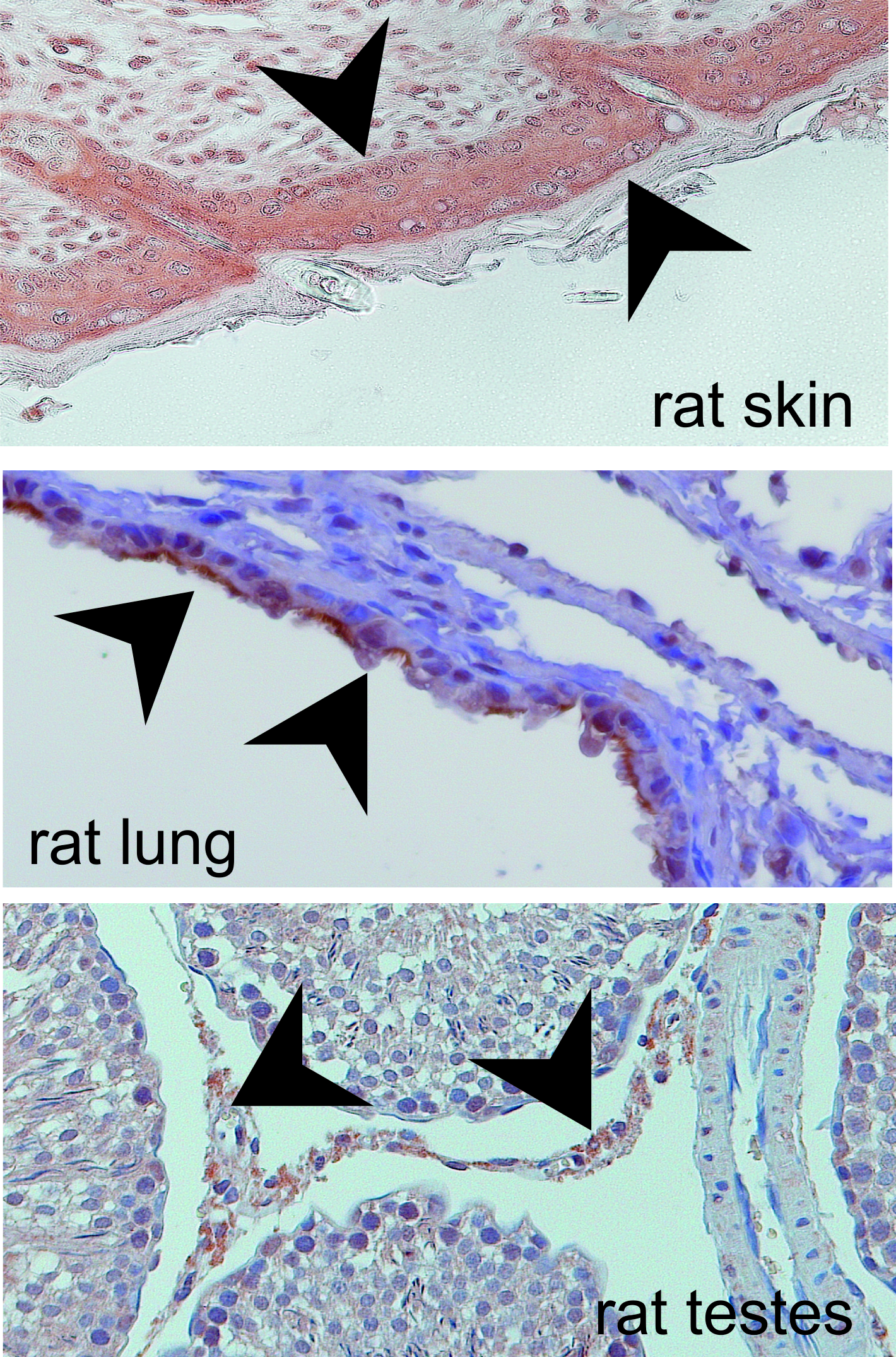

Supplement: Supplementary file 1 [file ijms-20-06240-s001.zip › Supplementary figure S6.tif]
